# A novel scoring schema for peptide identification by searching protein sequence databases using tandem mass spectrometry data

**DOI:** 10.1186/1471-2105-7-222

**Published:** 2006-04-26

**Authors:** Zhuo Zhang, Shiwei Sun, Xiaopeng Zhu, Suhua Chang, Xiaofei Liu, Chungong Yu, Dongbo Bu, Runsheng Chen

**Affiliations:** 1 Institute of Biophysics, Chinese Academy of Sciences, Beijing, P. R. China; 2 Institute of Computing Technology, Chinese Academy of Sciences, Beijing, P. R. China

## Abstract

**Background:**

Tandem mass spectrometry (MS/MS) is a powerful tool for protein identification. Although great efforts have been made in scoring the correlation between tandem mass spectra and an amino acid sequence database, improvements could be made in three aspects, including characterization ofpeaks in spectra, adoption of effective scoring functions and access to thereliability of matching between peptides and spectra.

**Results:**

A novel scoring function is presented, along with criteria to estimate the performance confidence of the function. Through learning the typesof product ions and the probability of generating them, a hypothetic spectrum was generated for each candidate peptide. Then relative entropy was introduced to measure the similarity between the hypothetic and the observed spectra. Based on the extreme value distribution (EVD) theory, a threshold was chosen to distinguish a true peptide assignment from a random one. Tests on a public MS/MS dataset demonstrated that this method performs better than the well-known SEQUEST.

**Conclusion:**

A reliable identification of proteins from the spectra promises a more efficient application of tandem mass spectrometry to proteomes with high complexity.

## Background

**Figure 1:**
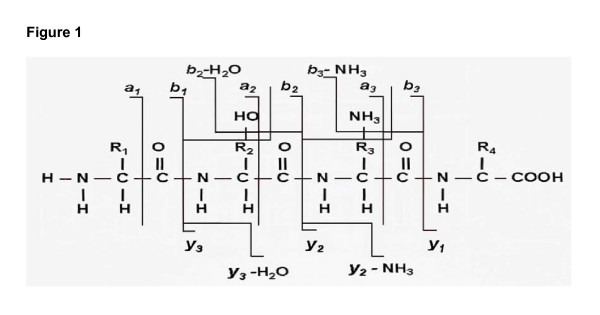
**Schematic figure of peptide fragmentation**. A peptide breaks into two parts, induced by the collisional gas. Breakage favors the peptide backbone, resulting in N-terminal a/b/c ions and C-terminal x/y/z/ions; small chemical groups such as water and amino molecules often dissociate from specific residues.

A major goal of proteomics is to study biological processes comprehensivelythrough the identification, characterization, and quantification of expressed protein in a cell or a tissue. Recently, tandem mass spectrometry has been shown to be a powerful tool for sensitive high-throughput identification of proteins [1,2]. Following enzymatic digestion of proteins, the resulting peptides are separated in the mass analyzer according to their mass to charge ratio (m/z-value). Peptides with the same m/z value are mostly brokeninto two fragments at a single peptide bond, forming N-terminal ions, i.e. *a*, *b or c*-ion and C-terminal ions, i.e., *x*, *y or z*-ion. All ions are then detected to generate a MS/MS spectrum (See Figure 1). In a mass spectrum, the intensity of a peak is generally proportional to the frequency of ions with the corresponding m/z-value [3].

A number of factors complicate the interpretation of MS/MS data. Neither the peaks corresponding to the *a*/*b*/*c *versus the *x*/*y*/*z *ions, nor the charge states of the ions are known. Some product fragments may be absent because either the fragments are in a neutral state or the precursorion hardly breaks down into these products; and unexplainable peaks may appear as the result of contamination or rare fragmentation styles. In addition, some peaks deviate from their expected positions because the corresponding ions contain isotopic atoms or have lost a chemical group. Consequently, effective identification of proteins remains a challenge [4].

Existing methods for interpreting mass spectrum data can becategorized into two types: database searching methods and '*de novo*' approaches independent of databases. Most database searching methods start with construction of a hypothetic spectrum for each peptide derived from a protein database, followed by comparing hypothetic and experimental spectra. The peptides with the highest score are reported as potential solutions. Employing this strategy, many systems have proven fairly successful [3,5,6].

An effective scoring function for evaluating matches between experimental spectrum and candidate peptide is a key issue in the interpretation of a mass spectrum. Most scoring functions, such as SEQUEST [5] and Sonar  are based on shared peaks. *p*-*value *is an alternative way to evaluate the probability of recognizing a set of fragments in a protein database, as implemented in MOWSE , Mascot [7] and ProbID [8]. Characterizing ion types and their probabilities, Dancik et al [9] proposed a likelihood-based approach, which was generalized in SCOPE [10] to involve more prior knowledge. An extension of Dancik's scoring approach into an intensity-based statistical scorer incorporated a variety of experimental observations and prior knowledge on peptide fragmentation [11]. ProbID [8], a method based on a probabilistic model, adopted a Bayesian approach to interpret mass spectra data.

Random matching between experimental and theoretical masses may bring aboutfalse-positive results, giving rise to another key problem of peptide identification---the criteria for evaluation of the reliability of the matching. The difference between the highest and second highest scores [5] and *p*-*value *[7] estimate are used to filter false positives. Jan Eriksson [12] and Keller [13] built a model to work out the distribution ofscores from random matches, which allowed significance testing undergeneral database searching constraints. Therefore, filtering criteria to distinguish a valid match from all matches should be developed towards being dependent on quantitative estimates rather than on experience.

The present paper aims to tackle the two problems mentioned above.

1. We introduce a new, effective probabilistic scoring function. Adopting astatistical model similar to Dancik et al [8], we have employedrelative entropy (i.e., K-L distance) to measure the similarity between hypothetic and experimentally observed spectra. We present a brief proof toshow that relative entropy is indeed the simplified form of the conditionalprobability that the spectrum is generated from the peptide.

2. We present an EVD-based criterion to distinguish valid matches from random ones. Each spectrum will acquire the best score from correlation with all candidate peptides. Such best scores conform to the extreme value distribution, which underlies the quantitative threshold of the significance test.

A system written in *C*, the Protein Identifier (*PI*), was implemented adopting the relative entropy scoring function and tested on real data. Tests showed that it performed better than a widely used cross-correlation scoring approach [5]. For the sake of convenience, we have made the program available on our website PI .

## Methods

### Data set

A publicly available spectrum set from *Keller et al *[14] was used to test our algorithm. It contains spectra generated by digesting two 18-proteins mixtures with trypsin. To assign a candidate peptide to each spectrum, Keller *et al *[14] ran SEQUEST against a human peptide database, obtaining 37,044 pairs of peptide and spectrum [14]. Among these assignments, 125 assignments of peptides with a single charge, 1,656 with double charges and 984 with triple charges were confirmed manually. For thefollowing two reasons, the original dataset above was subjected to a filtering procedure for refinement. First, because the enzyme had been slightly contaminated (private communication with A. Keller who published the data), peptides with abnormal termini were screened out. Second, the spectra from triply charged ions were filtered out because their fragmentation pattern is not yet fully understood. As for partial proteolysis, peptides with up to 5 trypsin cleavage sites were kept for database searching. The filtering procedure produced a refined query set containing 19,000 spectra, and 1,247 correct peptide assignments. Thereafter the 1,247 correct assignments was divided into a training set derived from 2 proteins and a test set derived from the other proteins for cross validation. The training set was used to study the characteristics of the mass spectrometry while the testing set was to test the performance of our algorithm. (Available at the website PI ).

### Description of algorithm

In order to describe our statistical model, a set of terms similar to Dancik et al [9] and Vineet Bafna et al [10] were defined as follows:

Let *A *be the set of amino acids, each amino acid having a molecular mass *m*(*a*), *a *∈ *A*. A peptide is denoted as a sequence of amino acids *P *= *p*_1_*p*_2_...*p*_*n *_with mass m(P)=∑im(pi)
 MathType@MTEF@5@5@+=feaafiart1ev1aaatCvAUfKttLearuWrP9MDH5MBPbIqV92AaeXatLxBI9gBaebbnrfifHhDYfgasaacH8akY=wiFfYdH8Gipec8Eeeu0xXdbba9frFj0=OqFfea0dXdd9vqai=hGuQ8kuc9pgc9s8qqaq=dirpe0xb9q8qiLsFr0=vr0=vr0dc8meaabaqaciaacaGaaeqabaqabeGadaaakeaacqWGTbqBdaqadaqaaiabdcfaqbGaayjkaiaawMcaaiabg2da9maaqafabaGaemyBa02aaeWaaeaacqWGWbaCdaWgaaWcbaGaemyAaKgabeaaaOGaayjkaiaawMcaaaWcbaGaemyAaKgabeqdcqGHris5aaaa@3B2B@, *p*_*i *_∈ *A*. Dissociation at the *i*-th peptide bond forms two partial peptides, the N-terminal peptide *P*_*i *_= *p*_1_*p*_2_...*p*_*i*_, *i *= 1,2,...,*n*, and the C-terminal peptide P′i
 MathType@MTEF@5@5@+=feaafiart1ev1aaatCvAUfKttLearuWrP9MDH5MBPbIqV92AaeXatLxBI9gBaebbnrfifHhDYfgasaacH8akY=wiFfYdH8Gipec8Eeeu0xXdbba9frFj0=OqFfea0dXdd9vqai=hGuQ8kuc9pgc9s8qqaq=dirpe0xb9q8qiLsFr0=vr0=vr0dc8meaabaqaciaacaGaaeqabaqabeGadaaakeaacuWGqbaugaqbamaaBaaaleaacqWGPbqAaeqaaaaa@2F68@ = *p*_*i*+1_...*p*_*n*_. Formally, ion types are represented as a set of numbers indicating the offset of ion to peptide, *δ *= {*δ*_1_,*δ*_2_...*δ*_*K*_}, thus, the i-type ion of the partial peptide *P*' has the mass *m*(*P*') + *δ*_*i*_. For example, *δ *= {1, -27, -17, -16} corresponds to the most frequent N-terminal ions, *b*, *a*, *b *- *H*_2_*O*, *b *-*NH*_3_, respectively. The interval from 0 to the precursor ion mass *M *= *m*(*P*) is discretized into *N *bins, hence, a MS/MS spectrum is represented as a vector of intensity, i.e., *S *= {*I*_1_,*I*_2_,...*I*_*N*_}, where *I*_*i *_is the intensity of the peak with the *m*/*z*-value i*M/*N*.

Fragmentation in mass spectrometry is a stochastic process governed by the collision dynamics and the physicochemical properties of a peptide [10]. The fragmentation of a set of the peptide in spectrometry was modeled by a random "ball tossing" trial, each trial tossing an N-terminal ion and/or a C-terminal ion in some bins. Thus, after a series of trials, the number of ions in a bin will be proportional to the intensity of the corresponding peak. The fragmentation of the different peptides, i.e., tossing of different balls, is assumed to be mutually independent. Let random variable *C *be the cleavage site, and Pr(*C *= *i*,*P*) be the probability of cleavage at the *i*-th bond of peptide *P*. Let Pr(*δ*_*i*_) be the probability that a breakage generates an *i*-type ion in a trial. Since fragmentation is residue dependent [15], we can adopt a more accurate definition, the conditional probability Pr(*δ*_*i*_|*p*_*j*_), where *p*_*j *_is the amino acid adjacent to the cleavage site. As mass spectrometry may also generate peaksdue to merely "random noise", Pr(*R*) is introduced to denote the probability of such event. For the sake of simplicity, only N-terminal ions are studied here; the considerations would have been similar for C-terminal ions.

### Parameter learning and hypothetic spectrum generating

To estimate the above peptide and instrument specific parameters, a learning procedure was modeled according to the offset frequency function [9], with improvements added to take account of intensity. For a set of experimental spectra with known peptide sequences thereof, let *S *= {*I*_1_,*I*_2_,...*I*_*N*_} be a spectrum corresponding to the peptide *P*, *ε *be the *m*/*z *precision level of the instrument, *N*(*S*,*P*_*i*_,*δ*_*j*_) be the sum of the peak intensities within a distance *ε *from *m*(*P*_*i*_) - *δ*_*j*_,*i *= 1,...*n*-1,*j *= 1,...|*δ*|, then N(S,Pi)=∑jN(S,Pi,δj)
 MathType@MTEF@5@5@+=feaafiart1ev1aaatCvAUfKttLearuWrP9MDH5MBPbIqV92AaeXatLxBI9gBaebbnrfifHhDYfgasaacH8akY=wiFfYdH8Gipec8Eeeu0xXdbba9frFj0=OqFfea0dXdd9vqai=hGuQ8kuc9pgc9s8qqaq=dirpe0xb9q8qiLsFr0=vr0=vr0dc8meaabaqaciaacaGaaeqabaqabeGadaaakeaacqWGobGtdaqadaqaaiabdofatjabcYcaSiabdcfaqnaaBaaaleaacqWGPbqAaeqaaaGccaGLOaGaayzkaaGaeyypa0ZaaabuaeaacqWGobGtdaqadaqaaiabdofatjabcYcaSiabdcfaqnaaBaaaleaacqWGPbqAaeqaaOGaeiilaWccciGae8hTdq2aaSbaaSqaaiabdQgaQbqabaaakiaawIcacaGLPaaaaSqaaiabdQgaQbqab0GaeyyeIuoaaaa@443F@ is the number of trials during which the *i*-th bond was dissociated. Dividing *N*(*S*,*P*_*i*_) by the total intensity, i.e., N(S,Pi)/∑jIj
 MathType@MTEF@5@5@+=feaafiart1ev1aaatCvAUfKttLearuWrP9MDH5MBPbIqV92AaeXatLxBI9gBaebbnrfifHhDYfgasaacH8akY=wiFfYdH8Gipec8Eeeu0xXdbba9frFj0=OqFfea0dXdd9vqai=hGuQ8kuc9pgc9s8qqaq=dirpe0xb9q8qiLsFr0=vr0=vr0dc8meaabaqaciaacaGaaeqabaqabeGadaaakeaacqWGobGtdaqadaqaaiabdofatjabcYcaSiabdcfaqnaaBaaaleaacqWGPbqAaeqaaaGccaGLOaGaayzkaaGaei4la8YaaabuaeaacqWGjbqsdaWgaaWcbaGaemOAaOgabeaaaeaacqWGQbGAaeqaniabggHiLdaaaa@3B22@, gives an estimate of Pr(*C *= *i*,*P*) over spectrum *S*, and *N*(*S*,*P*_*i*_,*δ*_*j*_)/*N*(*S*,*P*_*i*_) is a ditto of Pr(*δ*_*j*_). The probability of random noise Pr(*R*) is estimated as the sum of the intensities of the unexplained peaks divided bythe number of bins *N*. Averaging them over all samples will improve the estimation accuracy.

Fragmentation tends to occur more frequently in the middle than at ends of a peptide. J. Simpson [16] represented the relationship between peak's intensity and cleavage sites as a function of the relative position of a breakpoint, ranging from 0 to 1, whereas David L. Tabb[17] adopted a function based on relative mass of the partial peptide. Here, we used Tabb's approach to work out Pr(*C *= *i*,*P*).

Utilizing the above probability, we produced a hypothetic spectrum for a given peptide through simulating its dissociation in thespectrometry. A set of the peptide fragments at the *i*-th peptide bond with the probability Pr(*C *= *i*,*P*), and generates the *j*-type ion with probability Pr(*δ*_*j*_). Hence, peaks with mass *m*(*P*_*i*_) - *δ*_*j *_were assigned with relative intensity Pr(*C *= *i*,*P*)*Pr(*δ*_*j*_), and other peaks were assigned with Pr(*R*) to simulate random noises.

### Relative entropy based scoring function

Before comparison to the hypothetic spectrum, an experimental spectrum was pre-processed by dividing the intensity of each peak by the total ion intensity and filling blank bins with the value Pr(*R*) to simulate random noise. Hence, both the experimental and hypothetic spectra were transformed into distributions of ions over *N *bins, and relative entropy was employed to measure the similarity between them.

Relative entropy is a pertinent statistical tool to measure distance between two distributions [18]. For two distributions *U *= <*u*_1_,*u*_2_...*u*_*n*_>, ∑iui=1
 MathType@MTEF@5@5@+=feaafiart1ev1aaatCvAUfKttLearuWrP9MDH5MBPbIqV92AaeXatLxBI9gBaebbnrfifHhDYfgasaacH8akY=wiFfYdH8Gipec8Eeeu0xXdbba9frFj0=OqFfea0dXdd9vqai=hGuQ8kuc9pgc9s8qqaq=dirpe0xb9q8qiLsFr0=vr0=vr0dc8meaabaqaciaacaGaaeqabaqabeGadaaakeaadaaeqbqaaiabdwha1naaBaaaleaacqWGPbqAaeqaaOGaeyypa0JaeGymaedaleaacqWGPbqAaeqaniabggHiLdaaaa@3524@ and *V *= <*v*_*i*_,*v*_2_...*v*_*n*_>, ∑ivi=1
 MathType@MTEF@5@5@+=feaafiart1ev1aaatCvAUfKttLearuWrP9MDH5MBPbIqV92AaeXatLxBI9gBaebbnrfifHhDYfgasaacH8akY=wiFfYdH8Gipec8Eeeu0xXdbba9frFj0=OqFfea0dXdd9vqai=hGuQ8kuc9pgc9s8qqaq=dirpe0xb9q8qiLsFr0=vr0=vr0dc8meaabaqaciaacaGaaeqabaqabeGadaaakeaadaaeqbqaaiabdAha2naaBaaaleaacqWGPbqAaeqaaOGaeyypa0JaeGymaedaleaacqWGPbqAaeqaniabggHiLdaaaa@3526@, the relative entropy is defined as *H*(*U*,*V*) = ∑iui*ln⁡(ui/vi)
 MathType@MTEF@5@5@+=feaafiart1ev1aaatCvAUfKttLearuWrP9MDH5MBPbIqV92AaeXatLxBI9gBaebbnrfifHhDYfgasaacH8akY=wiFfYdH8Gipec8Eeeu0xXdbba9frFj0=OqFfea0dXdd9vqai=hGuQ8kuc9pgc9s8qqaq=dirpe0xb9q8qiLsFr0=vr0=vr0dc8meaabaqaciaacaGaaeqabaqabeGadaaakeaadaaeqbqaaiabdwha1naaBaaaleaacqWGPbqAaeqaaOGaeiOkaOIagiiBaWMaeiOBa42aaeWaaeaacqWG1bqDdaWgaaWcbaGaemyAaKgabeaakiabc+caViabdAha2naaBaaaleaacqWGPbqAaeqaaaGccaGLOaGaayzkaaaaleaacqWGPbqAaeqaniabggHiLdaaaa@3F49@. *H*(*U*,*V*) is always positive, and the more similar the two distributions, the smaller the relative entropy.

The relative entropy measure is essentially a simplified form of the likelihood Pr(*S*|*P*). Provided that *q*_*i *_is the probability that an ion will be tossed into the *i*-th bin, and *I*_*i *_the number of ions tossed into this bin after Itotal=∑iIi
 MathType@MTEF@5@5@+=feaafiart1ev1aaatCvAUfKttLearuWrP9MDH5MBPbIqV92AaeXatLxBI9gBaebbnrfifHhDYfgasaacH8akY=wiFfYdH8Gipec8Eeeu0xXdbba9frFj0=OqFfea0dXdd9vqai=hGuQ8kuc9pgc9s8qqaq=dirpe0xb9q8qiLsFr0=vr0=vr0dc8meaabaqaciaacaGaaeqabaqabeGadaaakeaacqWGjbqsdaWgaaWcbaGaemiDaqNaem4Ba8MaemiDaqNaemyyaeMaemiBaWgabeaakiabg2da9maaqafabaGaemysaK0aaSbaaSqaaiabdMgaPbqabaaabaGaemyAaKgabeqdcqGHris5aaaa@3C0D@ tosses, the count vector *I *= <*I*_1_,*I*_2_,...*I*_*N*_> conforms to a multi-nominal distribution. Hence, Pr(*S*|*P*) can be computed as follows: Pr⁡(S|P)=CItotalI1q1I1CItotal−I1I2q2I2……CItotal−I1−…−IN−1INqNIN
 MathType@MTEF@5@5@+=feaafiart1ev1aaatCvAUfKttLearuWrP9MDH5MBPbIqV92AaeXatLxBI9gBaebbnrfifHhDYfgasaacH8akY=wiFfYdH8Gipec8Eeeu0xXdbba9frFj0=OqFfea0dXdd9vqai=hGuQ8kuc9pgc9s8qqaq=dirpe0xb9q8qiLsFr0=vr0=vr0dc8meaabaqaciaacaGaaeqabaqabeGadaaakeaacyGGqbaucqGGYbGCdaqadaqaaiabdofatjabcYha8jabdcfaqbGaayjkaiaawMcaaiabg2da9iabdoeadnaaDaaaleaacqWGjbqsdaWgaaadbaGaemiDaqNaem4Ba8MaemiDaqNaemyyaeMaemiBaWgabeaaaSqaaiabdMeajnaaBaaameaacqaIXaqmaeqaaaaakiabdghaXnaaDaaaleaacqaIXaqmaeaacqWGjbqsdaWgaaadbaGaeGymaedabeaaaaGccqWGdbWqdaqhaaWcbaGaemysaK0aaSbaaWqaaiabdsha0jabd+gaVjabdsha0jabdggaHjabdYgaSbqabaWccqGHsislcqWGjbqsdaWgaaadbaGaeGymaedabeaaaSqaaiabdMeajnaaBaaameaacqaIYaGmaeqaaaaakiabdghaXnaaDaaaleaacqaIYaGmaeaacqWGjbqsdaWgaaadbaGaeGOmaidabeaaaaGccqWIMaYscqWIMaYscqWGdbWqdaqhaaWcbaGaemysaK0aaSbaaWqaaiabdsha0jabd+gaVjabdsha0jabdggaHjabdYgaSbqabaWccqGHsislcqWGjbqsdaWgaaadbaGaeGymaedabeaaliabgkHiTiablAciljabgkHiTiabdMeajnaaBaaameaacqWGobGtcqGHsislcqaIXaqmaeqaaaWcbaGaemysaK0aaSbaaWqaaiabd6eaobqabaaaaOGaemyCae3aa0baaSqaaiabd6eaobqaaiabdMeajnaaBaaameaacqWGobGtaeqaaaaaaaa@77E9@. For a spectrum, *I*_*i *_corresponds to the observed intensity of a peak with *m*/*z*-value *i*, where *I *is the vector form of a spectrum. Using Stirling's Formula, we can rewrite the above expression as follows:

Pr⁡(S|P)=CItotalI1q1I1CItotal−I1I2q2I2……CItotal−I1−…−IN−1INqNIN=Itotal!I1!……IN!∏i=1,…NqiIi≅2πItotal(Itotal/e)Itotal∏i=1,…N[2πIi(Ii/e)Ii]∏i=1,…NqiIi
 MathType@MTEF@5@5@+=feaafiart1ev1aaatCvAUfKttLearuWrP9MDH5MBPbIqV92AaeXatLxBI9gBaebbnrfifHhDYfgasaacH8akY=wiFfYdH8Gipec8Eeeu0xXdbba9frFj0=OqFfea0dXdd9vqai=hGuQ8kuc9pgc9s8qqaq=dirpe0xb9q8qiLsFr0=vr0=vr0dc8meaabaqaciaacaGaaeqabaqabeGadaaakqaaeeqaaiGbccfaqjabckhaYnaabmaabaGaem4uamLaeiiFaWNaemiuaafacaGLOaGaayzkaaGaeyypa0Jaem4qam0aa0baaSqaaiabdMeajnaaBaaameaacqWG0baDcqWGVbWBcqWG0baDcqWGHbqycqWGSbaBaeqaaaWcbaGaemysaK0aaSbaaWqaaiabigdaXaqabaaaaOGaemyCae3aa0baaSqaaiabigdaXaqaaiabdMeajnaaBaaameaacqaIXaqmaeqaaaaakiabdoeadnaaDaaaleaacqWGjbqsdaWgaaadbaGaemiDaqNaem4Ba8MaemiDaqNaemyyaeMaemiBaWgabeaaliabgkHiTiabdMeajnaaBaaameaacqaIXaqmaeqaaaWcbaGaemysaK0aaSbaaWqaaiabikdaYaqabaaaaOGaemyCae3aa0baaSqaaiabikdaYaqaaiabdMeajnaaBaaameaacqaIYaGmaeqaaaaakiablAciljablAciljabdoeadnaaDaaaleaacqWGjbqsdaWgaaadbaGaemiDaqNaem4Ba8MaemiDaqNaemyyaeMaemiBaWgabeaaliabgkHiTiabdMeajnaaBaaameaacqaIXaqmaeqaaSGaeyOeI0IaeSOjGSKaeyOeI0IaemysaK0aaSbaaWqaaiabd6eaojabgkHiTiabigdaXaqabaaaleaacqWGjbqsdaWgaaadbaGaemOta4eabeaaaaGccqWGXbqCdaqhaaWcbaGaemOta4eabaGaemysaK0aaSbaaWqaaiabd6eaobqabaaaaaGcbaGaeyypa0ZaaSaaaeaacqWGjbqsdaWgaaWcbaGaemiDaqNaem4Ba8MaemiDaqNaemyyaeMaemiBaWgabeaakiabcgcaHaqaaiabdMeajnaaBaaaleaacqaIXaqmaeqaaOGaeiyiaeIaeSOjGSKaeSOjGSKaemysaK0aaSbaaSqaaiabd6eaobqabaGccqGGHaqiaaWaaebuaeaacqWGXbqCdaqhaaWcbaGaemyAaKgabaGaemysaK0aaSbaaWqaaiabdMgaPbqabaaaaaWcbaGaemyAaKMaeyypa0JaeGymaeJaeiilaWIaeSOjGSKaemOta4eabeqdcqGHpis1aaGcbaGaeyyrIa0aaSaaaeaadaGcaaqaaiabikdaYGGaciab=b8aWjabdMeajnaaBaaaleaacqWG0baDcqWGVbWBcqWG0baDcqWGHbqycqWGSbaBaeqaaaqabaGcdaqadaqaaiabdMeajnaaBaaaleaacqWG0baDcqWGVbWBcqWG0baDcqWGHbqycqWGSbaBaeqaaOGaei4la8IaemyzaugacaGLOaGaayzkaaWaaWbaaSqabeaacqWGjbqsdaWgaaadbaGaemiDaqNaem4Ba8MaemiDaqNaemyyaeMaemiBaWgabeaaaaaakeaadaqeqbqaaiabcUfaBnaakaaabaGaeGOmaiJae8hWdaNaemysaK0aaSbaaSqaaiabdMgaPbqabaaabeaakmaabmaabaGaemysaK0aaSbaaSqaaiabdMgaPbqabaGccqGGVaWlcqWGLbqzaiaawIcacaGLPaaadaahaaWcbeqaaiabdMeajnaaBaaameaacqWGPbqAaeqaaaaakiabc2faDbWcbaGaemyAaKMaeyypa0JaeGymaeJaeiilaWIaeSOjGSKaemOta4eabeqdcqGHpis1aaaakmaarafabaGaemyCae3aa0baaSqaaiabdMgaPbqaaiabdMeajnaaBaaameaacqWGPbqAaeqaaaaaaSqaaiabdMgaPjabg2da9iabigdaXiabcYcaSiablAciljabd6eaobqab0Gaey4dIunaaaaa@E18A@

Hence ln[Pr(*S*|*P*)] ≅ ln*K *+ *I*_*total *_ln*I*_*total *_- *H*(*I*,*Q*), where *C *denotes combination and K=2πItotal∏i=1,…N2πIi
 MathType@MTEF@5@5@+=feaafiart1ev1aaatCvAUfKttLearuWrP9MDH5MBPbIqV92AaeXatLxBI9gBaebbnrfifHhDYfgasaacH8akY=wiFfYdH8Gipec8Eeeu0xXdbba9frFj0=OqFfea0dXdd9vqai=hGuQ8kuc9pgc9s8qqaq=dirpe0xb9q8qiLsFr0=vr0=vr0dc8meaabaqaciaacaGaaeqabaqabeGadaaakeaacqWGlbWscqGH9aqpdaWcaaqaamaakaaabaGaeGOmaidcciGae8hWdaNaemysaK0aaSbaaSqaaiabdsha0jabd+gaVjabdsha0jabdggaHjabdYgaSbqabaaabeaaaOqaamaarafabaWaaOaaaeaacqaIYaGmcqWFapaCcqWGjbqsdaWgaaWcbaGaemyAaKgabeaaaeqaaaqaaiabdMgaPjabg2da9iabigdaXiabcYcaSiablAciljabd6eaobqab0Gaey4dIunaaaaaaa@47C8@ embodies a characteristic of the spectrum. The above formula means that fora specific peptide, the smaller the relative entropy, the more likely that the spectrum was generated by that particular peptide.

### Threshold settings based on extreme value distribution

A score matrix was produced after comparing a set of spectra *S *= {*S*_1_,*S*_2_...*S*_*m*_} with a set of candidate peptides *C *= {*C*_1_,*C*_2_...*C*_*n*_} (Table. 1).

For a specific spectrum *S*_*i*_, the peptide with the minimal score was thought to be the one most likely to have generated it. The minimal score of a row, recorded in the "*min*" column, conforms to the extreme value distribution (EVD). In fact, the distribution of items in the *min *column is the superposition of two EVD distributions because of the following reason. Dividing the spectra in *S *into two classes, i.e. *S *= *D *∪ *E*, where *D *includes those that were generated from a peptidein *C*, and *E *the rest. Thus, the match of a spectrum with a peptide in *E *can occur only by chance, and therefore results in a minimal score higher than the matches with peptides in *D*. The gap between matches of the twopeptide sets, along with the robustness of EVD, forms a foundation to distinguish valid matches from random ones. More important, EVD allows us to quantitatively estimate the confidence for a given criterion. It should be noted that since the score of the peptides depends on the mass of the precursor ions, so does the threshold.

## Result and discussion

We performed the parameter learning, the hypothetic spectrum generation andthe search procedure on the chosen dataset as described above (See *Methods *section)

### Parameter learning

**Figure 2:**
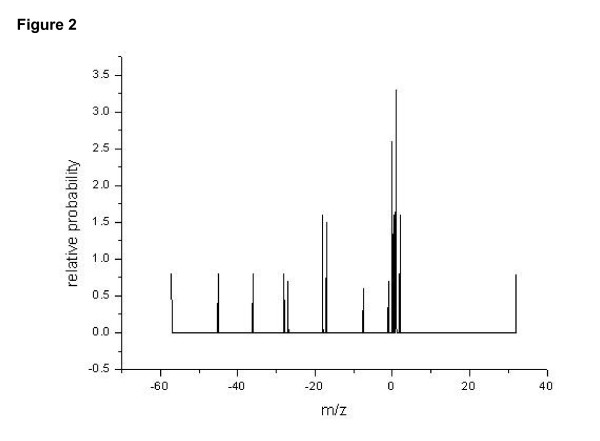
**Occurrence probability of different N-terminal ions**. In general, serial ions bear a larger probability, however, their variants (like dehydrated form) also often appear. The m/z of the peptide bond is set to 0, and the relative probability is normalized to a total probability of 100.0 (including C-terminal ions, which are not shown).

**Figure 3:**
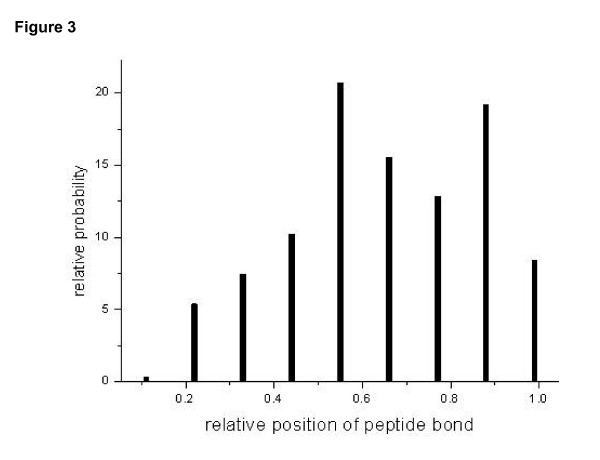
**Breakage preference in the position along peptides**. Breakage tends to occur at middle rather than terminal positions of the peptides. The Abscissa denotes the position of residues relative to the whole sequence. The relative probability is normalized to a total of 100.0.

For each round of test, the frequency of different ions were learnt from a training assignment comprising peptides from two to four proteins(generally less than 300 peptides assignments), including non-peptide bond backbone cleavage, isotope forms, and neutral loss (Figure 2). Here, the precision of an ion-trap mass spectrometry was set to 0.5 Dalton. It could beobserved that *b*, *a*, *b *- *H*_2_*O*, *b *- *NH*_3_, appear more frequently than other product ions, and isotope effect was also dominant. In Figure 3, the relationship between cleavage probability and relative position is shown as a bell-like curve. A conclusion could be made that cleavage tends to occur more frequently at middle than in the ends. The above observation coincides with the characteristic of fragmentation [11]. When the learning procedure was performed on different training sets, no obvious differences were observed for the learnt parameters.

### Hypothetic spectrum generation

**Figure 4:**
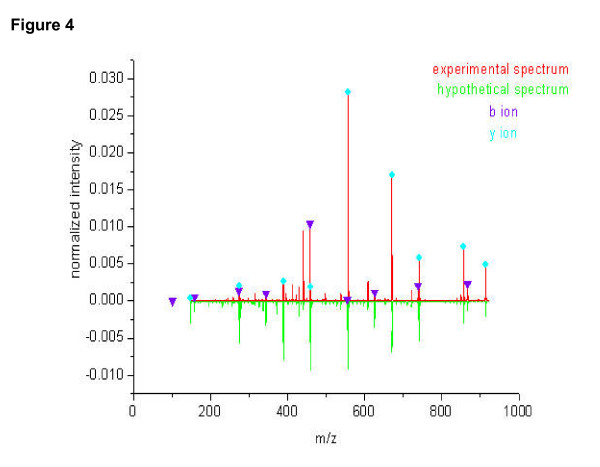
**The hypothetic and experimental spectra of the sequence *VGDANPALQK***. The serial ions are marked with the observed intensity.

To visualize the similarity and difference between hypothetic and experimental spectra, the hypothetic spectrum for a peptide *VGDANPALQK *was generated using the learnt parameters (shown in Figure 4 along with the experimental spectrum). As can be seen from the comparison, the hypothetic spectrum matches the experimental at important ions with respect to relative abundance, including serial ions *b*, *y*, andtheir isotope and group-loss forms (for the clarity of the figure, only b and y ion had been showed).

It should be pointed out that for the positions without hypothetic peaks, random noise learnt from training set was assigned to it. In fact, noise was an intrinsic feature for ion-trap mass spectrometry [19].

However, for some ions, there exists an obvious difference in the intensities between the two spectra. The main reasons for these discrepancies are the residues dependent cleavage and the complicated mobility patterns of the proton. To improve the accuracy of the learning procedure advanced learning techniques, such as EM methods, should be introduced in future work.

### Distribution of score and threshold setting

**Figure 5:**
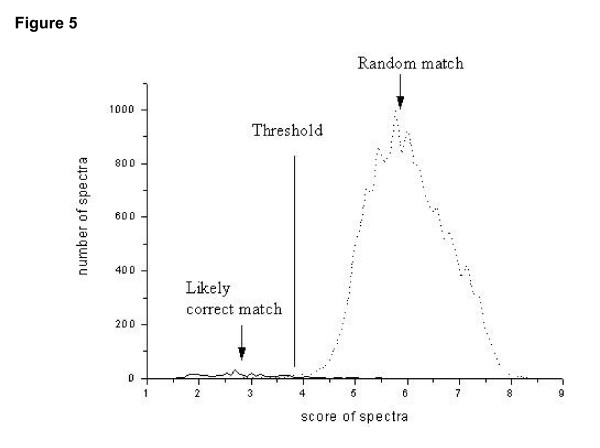
**Distribution of minimal score values of MS/MS spectra**. The score of a spectrum is the best match to the peptide library. The solidcurve results from correlating a set of MS/MS spectra with a peptide library containing the query peptide, while the dashed curve results from matcheswith a library not containing the query at all (i.e. all matches are random). Threshold is set to be the location of the cross-point of the two distributions, which minimizes the sum of false-positives and false-negatives. Therefore, if a match had a score less than 3.8, it was considered highly probable that the spectrum was produced as result of the matched peptide.

For each spectrum in a training set, similarity with all candidate peptidesare measured. The distribution of the best similarity gives a plot conforming to the extreme value distribution (Figure 5). For spectraderived from peptides with no counterpart in the candidate set, the score values are all generated by chance, and one peak with higher score is observed in diagram (Figure 5, dash line). However, for spectra that are derived from peptides with a counterpart in the candidate set, the likely correct matches between spectra and candidate peptides form a peak with lower score. Therefore, two peaks will be observed---one corresponding to the random matches, and the other to the likely correct matches, the latter with lower score values (Figure 5, solid curve). From Figure 5, it is clear that there is a distinct distance between the two peaks, which provides a basis for setting a threshold value to effectively distinguish correct matches from random ones. In our experiment, the threshold was set to 3.8, i.e., the location of the cross-point of the two distributions, which minimizes the sum of false-positives and false-negatives. Therefore, if a match had a score less than 3.8, it was considered highly probable that the spectrum was produced as result of the matched peptide. Threshold for three different training sets were 3.81, 3.66 and 4.09, respectively, that suggested the relative robustness of the threshold setting.

### Performance of the score function

Searching the query spectra against the same human peptide database as Keller *et al *[14] had been performed three times by selecting different proteins as testing sets. Take the first round as an example (Thefirst two proteins in the mixture were composed of the training set and therest test set). Our program *PI *reported 1,053 potential assignments, having 801 assignments overlap with the test set. For the 252 assignments inconsistent with the test set, manual inspection showed that 171 of them were correct. The difference in performance arose from SEQUEST neglecting some correct assignment, as reported by Andrew. Finally, *PI *achieved an average sensitivity of 0.87 and an average error rate of 0.07. Table 2 shows the comparison between the performances of SEQUEST and *PI*. As for the stability of our program, when *PI *was tested on a different dataset from OPD [20] it still displayed a sound performance (sensitivity 0.89 and error rate 0.06). It is interesting that performance of *PI *on OPD dataset was better than that on Keller's mixture dataset although the parameter training was done on the latter. This may be attributed to the different quality of these datasets. All the assignments are presented on our website at PI .

### Comparison of score functions

Roughly speaking, factors influencing the dissociation of peptides come in two categories. One is the chemical property of a peptide, and the other isthe collision energy. In recent years, much effort has been made to unravelthe fragmenting mechanism [15,21-26]. Based on of theoretical computation, experimental studies, and statistical analyses, models for prediction of product ions have become increasingly sophisticated and accurate, taking into consideration the chemistry of the ion rearrangements, gas-phase basicity of the individual residues, and the location effect. However, there is apparently still a way to go before the fragmentation mechanism is completely understood. Therefore, due to the incomplete understandingof the fragmentation chemistry, analyzers still have to rely on statisticalapproaches. A probabilistic formulation was originally brought forth by Dancik [9] and provoked several subsequent studies [10,27]. However, the principle of "a premium for present ions" and "a penalty for non-presentions" [9] aims only at the hypothetic but not at the experimentalspectrum. In other words, that formulation concentrates on what should appear in a hypothetic spectrum and neglects what emerges in the observed spectrum. Hence the formulation has the substantial defect that there is no penalty for emergence of unexplainable ions in an experimental spectrum. Our system describes the correlation between theoretical and experimental spectra in a bi-directional manner, taking account of non-coherence in a homogeneous way.

## Conclusion

In this paper we present a novel method for correlating peptides with tandem mass spectra. The collision-induced dissociation is analyzed as a random event and occurrence probabilities for characteristic ions are calculated. By using these parameters, a statistic model to predict the theoretical spectra for peptides is built. Based on the hypothetical spectra, the relative entropy is presented to correlate the experimental spectra with the hypothetical ones. Then it is pointed out that the score of spectra obeysthe superposition of two extreme value distributions, which allows the quantitative estimate for the confidence of peptide assignments. Computational experiment was done on two public databases and it showed that the performance of the present method was is superior in comparison with SEQUEST.

## Authors' contributions

R. S. Chen and D.B. Bu conceived and organized the research; D. B. Bu proposed the relative entropy scoring function and EVD-based threshold setting, and wrote the main part of program *PI*; Z. Zhang characterized the model of peptide fragmentation, carried out the computational study, analyzed the result and wrote the paper; S. W. Sun proposed the statistical model for theoretical spectrum prediction and learning procedure; X. P Zhu analyzed the characteristic of ions; S. H Chang helped Zhang test the program; X. F. Liu and C. G. Yu wrote the website and interface the of software.

## Figures and Tables

**Table 1:** An example of score table. Comparison of spectrum *S*_*i *_with candidate peptide *C*_*j *_produces a score in the matrix, and each item of the '*min*' column is the minimal score of the same row.

	*C*_1_	*C*_2_	...	*C*_*n*_	*min*
*S*_1_	2.4	3.7		8.9	2.4
*S*_2_	5.3	6.8		1.9	1.9
...					
*S*_*m*_	7.7	10.0		6.1	6.1

**Table 2:** Performance of SEQUEST and our program *PI*. SEQUEST achieved different sensitivities with different filtering criteria. Here the best one is listed.

	Sensitivity	Error rate
SEQUEST	0.78	0.09
*PI*	0.87	0.07
